# Supplementary material for: Scoping review on regulation, implementation and postmarket surveillance of medical devices
Source: PLoS One. 2025 May 30;20(5):e0325250. doi: 10.1371/journal.pone.0325250 (PMC12124561; doi:10.1371/journal.pone.0325250)
Supplement: S2 File — (DOCX) [file pone.0325250.s003.docx]

Appendix 2

**Supplementary table 1: Table over excluded studies**

| **Author (Year)** | **Title** | **Reason for exclusion** | **DOI-link or journal** |
| --- | --- | --- | --- |
| Abam 2020 | Prioritizing the Factors Affecting the Selection of Diagnostic Medical Equipment in Iranian Public Hospitals | Exclusion criteria: Diagnostic tests | Pakistan Journal of Medical and Health Sciences / 2020;14(2):1231-1235 |
| Abraham 2022 | FDA Reported Complications Associated With endoAVF Fistula Devices | Not generalizable | [Vascular Access: From Biology to Managing Complications](https://www.asn-online.org/education/kidneyweek/2022/program-abstract.aspx?controlId=3770926) |
| Abdulsalam 2021 | Of barriers and bridges: Buyer-supplier relationships in health care. | Does not fit PCC framework:  “*We carried out a systematic analysis of the data to reveal underlying factors that caused distrust and tensions between hospitals and manufacturers*” | <https://dx.doi.org/10.1097/HMR.0000000000000278> |
| Abel 2002 | Preclinical testing for aortic endovascular grafts: results of a Food and Drug Administration workshop. | Exclusion criteria: Opinion/Comment/Editorial | Journal of vascular surgery / 2002;35(5):1022-8 |
| Acosta 2022 | The Need for Medical Artificial Intelligence That Incorporates Prior Images. | Exclusion criteria: Opinion/Comment/Editorial | <https://dx.doi.org/10.1148/radiol.212830> |
| Ademe 2016 | Availability and utilization of medical devices in Jimma zone hospitals, Southwest Ethiopia: a case study. | Does not fit PCC framework: focus is on how devices works not how they were selected | <https://dx.doi.org/10.1186/s12913-016-1523-2> |
| Ahluwalia 2019 | Energy-based devices marketed for vaginal rejuvenation: An analysis of the maude database | Not generalizable | <https://dx.doi.org/10.1002/lsm.23059> |
| Allareddy 2017 | Adverse clinical events reported during Invisalign treatment: Analysis of the MAUDE database. | Not generalizable | <https://dx.doi.org/10.1016/j.ajodo.2017.06.014> |
| Almond 2012 | The FDA review process for cardiac medical devices in children: A review for the clinician | Not generalizable | <https://dx.doi.org/10.1016/j.ppedcard.2012.02.002> |
| Alonzo 2022 | Immunohematology and Blood Establishment Computer Software Medical Device Reports Submitted to US FDA in 2020 | Not generalizable | <https://dx.doi.org/10.1111/trf.17058> |
| Assam 2021 | Adverse event reporting in head and neck transoral robotic surgery: a MAUDE database study. | Not generalizable | <https://dx.doi.org/10.1007/s11701-020-01185-1> |
| Bahk 2015 | Increasing Patient Engagement in Pharmacovigilance Through Online Community Outreach and Mobile Reporting Applications: An Analysis of Adverse Event Reporting for the Essure Device in the US. | Not generalizable | Pharmaceutical medicine / 2015;29(6):331-340 |
| Bastani 2020 | Forty years review of upstream documents of the Islamic Republic of Iran's health sector on strategic purchasing of advanced-expensive medical equipment | Does not fit PCC framework:  "on strategic purchasing of advanced-expensive medical equipment" | <https://dx.doi.org/10.1108/IJHG-12-2019-0077> |
| Bayrak 2022 | What Will Be the Economic Impact of the New Medical Device Regulation? An Interrupted Time-Series Analysis of Foreign Trade Data. | Exclusion criteria: reimbursement | <https://dx.doi.org/10.1016/j.vhri.2021.07.010> |
| Beaussier 2012 | Medical devices used in interventional neuroradiology: Can we reach a clinical consensus to rationalize the number of references purchased in a French hospital group? | Not generalizable | <https://dx.doi.org/10.1007/s11096-011-9602-2> |
| Beck 2019 | Patient access to voice prostheses and heat and moisture exchangers: Factors influencing physician's prescription and reimbursement in eight European countries. | Does not fit PCC-framework | <https://dx.doi.org/10.1016/j.oraloncology.2019.02.017> |
| Beck 2019 | PMD25 FACTORS INFLUENCING PHYSICIAN'S PRESCRIPTION AND REIMBURSEMENT OF MEDICAL DEVICES USED IN LARYNGECTOMY REHABILITATION IN EUROPE | Does not fit PCC-framework | <https://dx.doi.org/10.1016/j.jval.2019.04.1022> |
| Billaux 2016 | Innovative medical devices and hospital decision making: a study comparing the views of hospital pharmacists and physicians. | Does not fit PCC-framework: more about satisfaction than underlying factors for decision-making according to PCC. | <https://dx.doi.org/10.1071/AH15039> |
| Bone 1995 | Standards of evidence for the safety and effectiveness of critical care monitoring devices and related interventions | Does not fit PCC-framework: Panel discussion | <https://dx.doi.org/10.1097/00003246-199510000-00022> |
| Borchers 2007 | The history and contemporary challenges of the US Food and Drug Administration | Exclusion criteria: Systematic review | Clinical therapeutics / 2007;29(1):1-16 |
| Boudard 2016 | [Introduction of Health technology assessment at hospital]. | Not generalizable, not enough information. | <https://dx.doi.org/10.1016/j.pharma.2016.03.001> |
| Bouschon 2019 | PMD37 EVOLUTION OF UNION EUROPEAN MEDICAL DEVICE REGULATION: PERSPECTIVE WITH THE CLINICAL EVALUATION OF DUAL MOBILITY ACETABULAR CUPS BY THE FRENCH NATIONAL HEALTH TECHNOLOGY ASSESSMENT AGENCY | Does not fit PCC-framework: Abstract only, no relevant data. | <https://dx.doi.org/10.1016/j.jval.2019.09.1450> |
| Brown 2004 | Medical device epidemiology and surveillance: Patient safety is the bottom line | Exclusion criteria: Opinion/Comment/Editorial | <https://dx.doi.org/10.1586/17434440.1.1.1> |
| Bucciarelli 2022 | Use of Bombyx mori silk fibroin in tissue engineering: From cocoons to medical devices, challenges, and future perspectives. | Does not fit PCC-framework | <https://dx.doi.org/10.1016/j.bioadv.2022.212982> |
| Buechel 1991 | Use of survivorship and contact stress analyses to predict the long-term efficacy of new generation joint replacement designs. A model for FDA device evaluation. | Exclusion criteria: Opinion/Comment/Editorial | Orthopaedic review / 1991;20(1):50-5 |
| Buescher 2017 | High battery impedance in the pacemaker dependent patient: Early recognition and collaborative management of insync III CRT-P device failures | Not generalizable: Battery Impedance in pacemakers too narrow. | <https://dx.doi.org/10.1016/S0735-1097> |
| Burns 2017 | Evidence gathering across key stakeholders involved in early health technology assessment | Does not fit PCC-framework | <https://dx.doi.org/10.1017/S026646231700126X> |
| Burton 2018 | Industry And Clinician Views Of Medtech Innovation Briefings | Does not fit PCC-framework | <https://dx.doi.org/10.1017/S0266462318001630> |
| Campbell 2017 | Public consultation changes guidance on the use of health-care interventions. An observational study. | Does not fit PCC-framework: reply to guidelines, does not fit our PCC implementation domain definition | <https://dx.doi.org/10.1111/hex.12476> |
| Campbell 2018 | ASSESSING THE VALUE OF INNOVATIVE MEDICAL DEVICES AND DIAGNOSTICS: THE IMPORTANCE OF CLEAR AND RELEVANT CLAIMS OF BENEFIT. | Does not fit PCC-framework | <https://dx.doi.org/10.1017/S0266462318000466> |
| Cao 2022 | Development of a Supplier Scorecard for a Medical Device Company to Formalize Its Supplier Monitoring and Re-evaluation Process Toward ISO13485:2016 | Does not fit PCC-framework: Structural organizing not implementation definition | <https://dx.doi.org/10.1007/s12247-021-09604-y> |
| Carlton 2022 | UNDERSTANDING TWO DECADES OF SAFETY REPORTING FOR SACRONEUROMODULATION: AN ANALYSIS OF THE FDA MAUDE DATABASE | Not generalizable | <https://dx.doi.org/10.1097/SPV.0000000000001202> |
| Carlton 2020 | New Medical Device Acquisition During Pediatric Severe Sepsis Hospitalizations. | Does not fit PCC-framework: more devices but still very narrow with one outcome | <https://dx.doi.org/10.1097/CCM.0000000000004272> |
| Cavanaugh 2018 | Harmonization by Doing Proposal for Global Clinical Trial Designs for Endovascular Devices for Treatment of Critical Limb Ischemia: The United States Food and Drug Administration Perspective. | Exclusion criteria:  Opinion/Comment/Editorial | <https://dx.doi.org/10.1253/circj.CJ-18-0817> |
| Chambers 2013 | Medicare covers the majority of FDA-approved devices and part B drugs, but restrictions and discrepancies remain | Exclusion criteria: Reimbursement | <https://dx.doi.org/10.1377/hlthaff.2012.1073> |
| Chen 2019 | PSU32 ECONOMIC ANALYSIS OF NEW ULTRASONIC ENERGY DEVICEi1/4sFROM HOSPITAL PERSPECTIVE | Does not fit PCC-framework: Financial analysis | <https://dx.doi.org/10.1016/j.jval.2019.04.1812> |
| Childerhose 2013 | Health consumption as work: The home pregnancy test as a domesticated health tool | Exclusion criteria: Diagnostic test | <https://dx.doi.org/10.1016/j.socscimed.2013.02.035> |
| Choudhury 2019 | Modern medical devices and hospital decision-making: A review of ethical and social considerations during the manufacture and the use of advanced medical devices | Exclusion criteria: Systematic review | <https://dx.doi.org/10.22452/jummec.vol22no1.1> |
| Ciani 2015 | Health technology assessment of medical devices: a survey of non-European union agencies. | Does not fit PCC-framework | <https://dx.doi.org/10.1017/S0266462315000185> |
| Cirkovic 2020 | Evaluation of Four Artificial Intelligence-Assisted Self-Diagnosis Apps on Three Diagnoses: Two-Year Follow-Up Study. | Exclusion criteria: Artificial intelligence | <https://dx.doi.org/10.2196/18097> |
| Coroneos 2019 | US FDA Breast Implant Postapproval Studies: Long-term Outcomes in 99,993 Patients. | Not generalizable | <https://dx.doi.org/10.1097/SLA.0000000000002990> |
| Couto 2022 | Unique Device Identifiers for Cardiac Implantable Electronic Devices - A Data Quality Assessment of Administrative Hospital Data in Portugal | Does not fit PCC-framework | <https://dx.doi.org/10.1016/j.jval.2022.09.2422> |
| Daubner-Bendes 2020 | Quo Vadis HTA for Medical Devices in Central and Eastern Europe? Recommendations to Address Methodological Challenges. | Exclusion criteria: Systematic review | <https://dx.doi.org/10.3389/fpubh.2020.612410> |
| Darrow 2021 | FDA Regulation and Approval of Medical Devices: 1976-2020 | Do not fit PCC-framework | <https://dx.doi.org/10.1001/jama.2021.11171> |
| Deininger 2019 | Prototyping for context: exploring stakeholder feedback based on prototype type, stakeholder group and question type. | Does not fit PCC-framework | <https://dx.doi.org/10.1007/s00163-019-00317-5> |
| Dhruva 2019 | Using the va national cardiac device surveillance program to monitor the safety of cardiovascular implantable electronic devices | Does not fit PCC-framework | <https://dx.doi.org/10.1161/hcq.12.suppl-1.119> |
| Dhruva 2022 | Strategies to Manage Drugs and Devices Approved Based on Limited Evidence: Results of a Modified Delphi Panel. | Does not fit PCC-framework | <https://dx.doi.org/10.1002/cpt.2583> |
| Dhruva 2023a | Using real-world data from health systems to evaluate the safety and effectiveness of a catheter to treat ischemic ventricular tachycardia | Not generalizable | <https://dx.doi.org/10.1007/s10840-023-01496-x> |
| Dhruva 2023b | Exploring unique device identifier implementation and use for real-world evidence: a mixed-methods study with NESTcc health system network collaborators | Does not fit PCC-framework | <https://dx.doi.org/10.1136/bmjsit-2022-000167> |
| DiLascio 2020 | The cis/pacs system: Experience in the hemodynamics department | Does not fit PCC-framework | <https://dx.doi.org/10.1093/eurheartj/suaa106> |
| Diani 2017 | An evaluation of the effectiveness of a risk-based monitoring approach implemented with clinical trials involving implantable cardiac medical devices. | Does not fit PCC-framework | <https://dx.doi.org/10.1177/1740774517723589> |
| Droschel 2017 | Integrated care models in Germany-potential market access route for medical devices? | Exclusion criteria: Reimbursement | <https://dx.doi.org/10.1016/j.jval.2017.08.623> |
| Drozda 2018 | Constructing the informatics and information technology foundations of a medical device evaluation system: a report from the FDA unique device identifier demonstration. | Does not fit PCC-framework | <https://dx.doi.org/10.1093/jamia/ocx041> |
| Dubin 2022 | The FDA and Ensuring Safety and Effectiveness of Devices, Biologics, and Technology | Exclusion criteria: Systematic review | <https://dx.doi.org/10.5435/JAAOS-D-22-00179> |
| Duggirala 2012 | Disproportionality analysis for signal detection of implantable cardioverter-defibrillator-related adverse events in the Food and Drug Administration Medical Device Reporting System. | Not generalizable | <https://dx.doi.org/10.1002/pds.2261> |
| Dutot 2017 | HOSPITAL-BASED HEALTH TECHNOLOGY ASSESSMENT FOR THE ADOPTION OF INNOVATIVE MEDICAL DEVICES WITHIN FRENCH HOSPITALS: OPPORTUNITIES AND CHALLENGES FOR INDUSTRY | Not generalizable | <https://dx.doi.org/10.1017/S0266462317000368> |
| Dvir 2012 | Overview of the 2011 food and drug administration's circulatory system devices panel of the medical devices advisory committee meeting on the Zilver R PTX R drug-eluting peripheral stent. | Not generalizable | <https://dx.doi.org/10.1016/j.carrev.2012.05.003> |
| Eikelboom 1996 | Regulatory requirements for medical devices in the European Union. | Exclusion criteria: Editorial/Opinion/Comment paper | European journal of vascular and endovascular surgery : the official journal of the European Society for Vascular Surgery / 1996;12(1):3-4 |
| Esser 2020 | PNS100 The New Reimbursement Route for Digital Health Applications (DIGA) in Germany: Critical Appraisal and First Evaluation of the Possible Effect on the German Healthcare System | Exclusion criteria: Reimbursement | <https://dx.doi.org/10.1016/j.jval.2020.08.1544> |
| Farrah 2019 | Almost half of references in reports on new and emerging nondrug health technologies are grey literature. | Does not fit PCC-framework | <https://dx.doi.org/10.5195/jmla.2019.539> |
| Federici 2021 | Coverage with evidence development schemes for medical devices in Europe: characteristics and challenges. | Exclusion criteria: Reimbursement | <https://dx.doi.org/10.1007/s10198-021-01334-9> |
| Feldman 2008 | Who is responsible for evaluating the safety and effectiveness of medical devices? The role of independent technology assessment | Exclusion criteria: Editorial/Opinion/Comment paper | <https://dx.doi.org/10.1007/s11606-007-0275-4> |
| Fradet 2018 | HOSPITAL-BASED HEALTH TECHNOLOGY ASSESSMENT OF MEDICAL DEVICES IN FRANCE: A COLLABORATION WITH MEDICAL DEVICE MANUFACTURERS OR NATIONAL AGENCY? | Does not fit PCC-framework | <https://dx.doi.org/10.1016/j.jval.2018.09.1621> |
| Fuchs 2019 | Testing a new taxonomic model for the assessment of medical devices: Is it plausible and applicable? Insights from HTA reports and interviews with HTA institutions in Europe. | Does not fit PCC-framework | <https://dx.doi.org/10.1016/j.healthpol.2018.03.004> |
| Fuller 2005 | Trocar-associated injuries and fatalities: an analysis of 1399 reports to the FDA. | Not generalizable | Journal of minimally invasive gynecology / 2005;12(4):302-7 |
| Gagliardi 2017 | Factors constraining patient engagement in implantable medical device discussions and decisions: interviews with physicians. | Does not fit PCC-framework | <https://dx.doi.org/10.1093/intqhc/mzx013> |
| Gamessa 2022 | Planning and Budgeting of Medical Devices Among Ethiopian Public Hospitals. | Does not fit PCC-framework: Organization not implementation | <https://dx.doi.org/10.2147/CEOR.S363376> |
| Garver 1995 | The epidemiology of prosthetic heart valves in the United States. | Not generalizable | Texas Heart Institute journal / 1995;22(1):86-91 |
| Gavaghan 2013 | Health economic modeling methods from an ACO provider perspective | Exclusion criteria: Reimbursement | <https://dx.doi.org/10.1016/j.jval.2013.03.164> |
| Ghobadi 2017 | Radiological Medical Device Innovation: Approvals via the Premarket Approval Pathway From 2000 to 2015. | Not generalizable | <https://dx.doi.org/10.1016/j.jacr.2016.08.014> |
| Glen 2018 | THE USE OF MEDTECH INNOVATION BRIEFINGS (MIBS) TO ENCOURAGE IMPLEMENTATION OF INNOVATIVE TECHNOLOGIES: A REVIEW OF PUBLISHED BRIEFINGS FROM 2014-2018 | Does not fit PCC-framework | <https://dx.doi.org/10.1016/j.jval.2018.09.1087> |
| Golish 2017 | Spinal devices in the United States-investigational device exemption trials and premarket approval of class III devices. | Not generalizable | <https://dx.doi.org/10.1016/j.spinee.2016.09.015> |
| Grandeau 2015 | Traceability of implantable medical devices: A retrospective study from 2010 to 2013 | Does not fit PCC-framework | <https://dx.doi.org/10.1136/ejhpharm-2015-000639.485> |
| Green 2020 | MT3 A Review of the National Institute for Health and Care Excellence (NICE) Medical Technologies Guidance over the Last 10 YEARS | Not generalizable | <https://dx.doi.org/10.1016/j.jval.2020.08.053> |
| Griffiths 2017 | Undermining the gold standard? the role of non-comparative evidence in health technology assessment | Does not fit PCC-framework | Value in Health / 2017;20(5):A20 |
| Grigore 2020 | Surrogate Endpoints in Health Technology Assessment: An International Review of Methodological Guidelines | Does not fit PCC-framework | <https://dx.doi.org/10.1007/s40273-020-00935-1> |
| Gunasekeran 2022 | Acceptance and Perception of Artificial Intelligence Usability in Eye Care (APPRAISE) for Ophthalmologists: A Multinational Perspective. | Exclusion criteria: Opinion/Survey | <https://dx.doi.org/10.3389/fmed.2022.875242> |
| Haas 2017 | Drivers of the Variation in Prosthetic Implant Purchase Prices for Total Knee and Total Hip Arthroplasties. | Exclusion criteria: Reimbursement | <https://dx.doi.org/10.1016/j.arth.2016.07.030> |
| Harris 2009 | FDA regulation of clinical high intensity focused ultrasound (HIFU) devices. | Exclusion criteria: Editorial/Opinion/Comment paper | <https://dx.doi.org/10.1109/IEMBS.2009.5332444> |
| Hassanin 2023 | Image-Guided Surgical Device Failures in Functional Endoscopic Sinus Surgery: A MAUDE Analysis. | Not generalizable | <https://dx.doi.org/10.1002/lary.30296> |
| Henein 2020 | Fifteen-year analysis of FDA recalls of ophthalmic implants | Not generalizable | Investigative Ophthalmology and Visual Science / 2020;61(7) |
| Hennein 2022 | Stakeholder perspectives on scaling up medical device reprocessing: A qualitative study. | Does not fit PCC-framework: The aim of the paper is repair of devices | <https://dx.doi.org/10.1371/journal.pone.0279808> |
| Higa 2020 | Risk-Based Monitoring Approach to Ensure the Quality of Clinical Study Data and Enable Effective Monitoring. | Does not fit PCC-framework | <https://dx.doi.org/10.1007/s43441-019-00037-x> |
| Hinrichs 2010 | A case study of design methods applied to researching medical device purchasing processes | Does not fit PCC-framework | <https://dx.doi.org/10.4066/AMJ.2010.410> |
| Hoogendoorn 2023 | What Makes a Quality Health App-Developing a Global Research-Based Health App Quality Assessment Framework for CEN-ISO/TS 82304-2: Delphi Study. | Does not fit PCC-framework: Quality standards for app, not related to approval. | <https://dx.doi.org/10.2196/43905> |
| Hristova-Neeley 2015 | The rise of the value analysis committee at us hospitals, better or worse for medical device companies? | Does not fit PCC-framework | Value in Health / 2015;18(3):A49 |
| Hsi 2009 | Analysis of techniques to secure the renal hilum during laparoscopic donor nephrectomy: review of the FDA database. | Not generalizable | <https://dx.doi.org/10.1016/j.urology.2008.11.010> |
| Hua 2016 | Transcatheter closure of perimembranous ventricular septal defects with the Amplatzer Vascular Plug-II. | Not generalizable | <https://dx.doi.org/10.1017/S1047951115002206> |
| Huijghebaert 2021 | Does trypsin oral spray (Viruprotect / coldzyme ) protect against covid-19 and common colds or induce mutation? caveats in medical device regulations in the european union | Not generalizable | <https://dx.doi.org/10.3390/ijerph18105066> |
| Hung 2022 | Regulations on Purchasing Medical Equipment and Investing Technology and Equipment for Cancer Treatment at Bai Chay Hospital, Quang Ninh | Does not fit PCC-framework | <https://dx.doi.org/10.47750/pnr.2022.13.S03.004> |
| Huusko 2023 | Medical device regulation (MDR) in health technology enterprises - perspectives of managers and regulatory professionals. | Does not fit PCC-framework: Questionnaire about law (MDR) | <https://dx.doi.org/10.1186/s12913-023-09316-8> |
| Imaz-Iglesia 2021 | Post-Launch Evidence Generation Studies For Medical Devices In Spain: Integrating Real World Evidence Into Decision-Making | Does not fit PCC-framework | <https://dx.doi.org/10.1017/S0266462321000751> |
| Izumi 2004 | Development of a tissue-engineered human oral mucosa: from the bench to the bed side. | Not generalizable | Cells, tissues, organs / 2004;176(1-3):134-52 |
| Jacobs 2017 | DEFINING THE RELEVANT OUTCOME MEASURES IN MEDICAL DEVICE ASSESSMENTS: AN ANALYSIS OF THE DEFINITION PROCESS IN HEALTH TECHNOLOGY ASSESSMENT. | Does not fit PCC-framework | <https://dx.doi.org/10.1017/S0266462317000216> |
| Jairam 2017 | Predictors of wrong dose, volume, or targeting events within fda recalls of linear accelerators | Not generalizable | <https://dx.doi.org/10.1016/j.ijrobp.2017.06.175> |
| Johnson 2007 | Attitudes toward medical device use errors and the prevention of adverse events. | Does not fit PCC-framework | Joint Commission journal on quality and patient safety / 2007;33(11):689-94 |
| Jones 2018 | Registry Assessment of Peripheral Interventional Devices (RAPID): Registry assessment of peripheral interventional devices core data elements. | Does not fit PCC-framework | <https://dx.doi.org/10.1016/j.jvs.2017.07.141> |
| Jorgensen 2017 | Patient participation is crucial when introducing new device technologies in the management of chronic arthritis: Applying the parker model, a qualitative 3-step approach | Does not fit PCC-framework | <https://dx.doi.org/10.1136/annrheumdis-2017-eular.1770> |
| Jorgensen 2018 | The Parker Model: Applying a Qualitative Three-Step Approach to Optimally Utilize Input from Stakeholders When Introducing New Device Technologies in the Management of Chronic Rheumatic Diseases. | Does not fit PCC-framework | <https://dx.doi.org/10.1007/s40271-018-0306-8> |
| Judson 2017 | Evaluation of cardiovascular devices approved for medicare new technology addon payments | Not generalizable | Circulation / 2017;136 |
| Kabeta 2023 | Medical Equipment Management in General Hospitals: Experience of Tulu Bolo General Hospital, South West Shoa Zone, Central Ethiopia. | Not generalizable | <https://dx.doi.org/10.2147/MDER.S398933> |
| Kadakia 2022 | Renewing the Call for Reforms to Medical Device Safety-The Case of Penumbra. | Not generalizable | <https://dx.doi.org/10.1001/jamainternmed.2021.6626> |
| Kaplan 2004 | Medical device development: from prototype to regulatory approval. | Exclusion criteria: Editorial/Opinion/Comment paper | Circulation / 2004;109(25):3068-72 |
| Karekar 2021 | Current status of clinical research using artificial intelligence techniques: A registry-based audit. | Does not fit PCC-framework | <https://dx.doi.org/10.4103/picr.PICR_25_20> |
| Katz 2018 | Adverse events and device malfunctions associated with spinal cord stimulators: Analysis of FDA MAUDE database | Not generalizable | <https://dx.doi.org/10.1111/ner.12774> |
| Kayser 2018 | Prevalence and Analysis of Medical Device-Related Pressure Injuries: Results from the International Pressure Ulcer Prevalence Survey. | Class I / Not generalizable | <https://dx.doi.org/10.1097/01.ASW.0000532475.11971.aa> |
| Kendzerska 2016 | The Effect of Patient Neighborhood Income Level on the Purchase of Continuous Positive Airway Pressure Treatment among Patients with Sleep Apnea. | Does not fit PCC-framework | <https://dx.doi.org/10.1513/AnnalsATS.201505-294OC> |
| Khalid 2019 | Adverse Events and Modes of Failure Related to Impella RP: A Retrospective Analysis of the Manufacturer and User Facility Device Experience (MAUDE) Database | Not generalizable | <https://dx.doi.org/10.1016/j.jcin.2019.01.147> |
| Khan 2017 | Stent thrombosis with bioabsorbable and second generation durable polymer platforms: Insights from the FDA manufacturer and user facility device experience (MAUDE) Database | Not generalizable | <https://dx.doi.org/10.1002/ccd.27053> |
| Kheir 2022 | Risk Identification and Analysis in the Development of Medical Devices Among Start-Ups: Towards a Broader Risk Management Framework | Does not fit PCC-framework | <https://dx.doi.org/10.2147/MDER.S375977> |
| Khunte 2023 | Trends in clinical validation and usage of US Food and Drug Administration-cleared artificial intelligence algorithms for medical imaging. | Does not fit PCC-framework | <https://dx.doi.org/10.1016/j.crad.2022.09.122> |
| Kikuchi 2021 | The Significance of Alliance Networks in Research and Development of Digital Health Products for Diabetes: Observational Study. | Does not fit PCC-framework: The paper is about collaboration and development of digital health products | <https://dx.doi.org/10.2196/32446> |
| Kisser 2016 | Factors influencing coverage decisions on medical devices: A retrospective analysis of 78 medical device appraisals for the Austrian hospital benefit catalogue 2008-2015. | Exclusion criteria: Reimbursement | <https://dx.doi.org/10.1016/j.healthpol.2016.06.007> |
| Klein 2022 | Real-world evidence in health technology assessment of high-risk medical devices: Fit for purpose? | Does not fit PCC-framework | <https://dx.doi.org/10.1002/hec.4575> |
| Klonoff 2018 | Investigation of the Accuracy of 18 Marketed Blood Glucose Monitors. | Not generalizable | <https://dx.doi.org/10.2337/dc17-1960> |
| Koldeweij 2021 | CE Accreditation and Barriers to CE Marking of Pediatric Drug Calculators for Mobile Devices: Scoping Review and Qualitative Analysis. | Does not fit PCC-framework: Not FDA/EU approved | <https://dx.doi.org/10.2196/31333> |
| Koutsogiannis 2021 | A Cross-sectional Analysis of 284 Complications for Lumbar Disc Replacements from Medical Device Reports Maintained by the United States Food and Drug Administration | Not generalizable | <https://dx.doi.org/10.1016/j.spinee.2021.08.001> |
| Kovacs 2021 | PNS77 Transferring Health Technology Assessments from EARLY Adopter to Late Adopter Countries: Recommendations for the Implementation of CED Schemes in Central and Eastern European Countries | Not generalizable | <https://dx.doi.org/10.1016/j.jval.2021.04.931> |
| Kramer 2023 | Quantitative Analyses of Regulatory Policies for Medical Devices: Matching the Methods to the Moment | Exclusion criteria: Editorial/Opinion/Comment paper | <https://dx.doi.org/10.1001/jama.2022.23888> |
| Kumar 2023 | Medtech Innovations in Oncology: A Review of NICE Medtech Innovation Briefings from 2014 to 2022 | Does not fit PCC-framework | <https://dx.doi.org/10.1016/j.jval.2023.03.1676> |
| Lawal 2018 | The nature, magnitude, and reporting compliance of device-related events for intravenous patient-controlled analgesia in the FDA Manufacturer and User Facility Device Experience (MAUDE) database. | Not generalizable | <https://dx.doi.org/10.1080/14740338.2018.1442431> |
| Lema 2022 | Post-Launch Evidence Generation Among Health Technology Assessment Bodies In Europe | Does not fit PCC-framework: | <https://dx.doi.org/10.1017/S0266462322000691> |
| Lingg 2016 | How does the knowledge environment shape procurement practices for orthopaedic medical devices in Mexico?. | Does not fit PCC-framework | <https://dx.doi.org/10.1186/s12911-016-0324-1> |
| Liu 2017 | Food and Drug Administration's Humanitarian Device Exemption marketing approval pathway: Insights for developing devices for rare diseases | Exclusion criteria: Editorial/Opinion/Comment paper | <https://dx.doi.org/10.1115/1.4036333> |
| Livingston 2021 | Drug and medical device product failures and the stability of the pharmaceutical supply chain. | Does not fit PCC-framework: Focus is more on drugs | <https://dx.doi.org/10.1016/j.japh.2020.07.005> |
| Long 2022 | Developing minimum core data structure for the obesity devices Coordinated Registry Network (CRN). | Does not fit PCC-framework | <https://dx.doi.org/10.1136/bmjsit-2021-000118> |
| Long 2014 | Evolving provider payment models and patient access to innovative medical technology | Exclusion criteria: Reimbursement | <https://dx.doi.org/10.3111/13696998.2014.965255> |
| Lu 2015 | Tissue engineered constructs: perspectives on clinical translation. | Exclusion criteria: Editorial/Opinion/Comment paper | <https://dx.doi.org/10.1007/s10439-015-1280-0> |
| Lueza 2022 | MT8 A Systematic Literature Review of Published Economic Evaluations for Medical Devices in Cardiovascular Disease | Exclusion criteria: Systematic review | <https://dx.doi.org/10.1016/j.jval.2022.09.1882> |
| Lubbeke 2023 | Clinical investigations to evaluate high-risk orthopaedic devices: a systematic review of the peer-reviewed medical literature | Exclusion criteria: Systematic review | <https://dx.doi.org/10.1530/EOR-23-0024> |
| Maas 2022 | The Definitions of Health Apps and Medical Apps From the Perspective of Public Health and Law: Qualitative Analysis of an Interdisciplinary Literature Overview. | Exclusion criteria: Systematic review | <https://dx.doi.org/10.2196/37980> |
| Mackintosh 2018 | Rethinking health sector procurement as developmental linkages in East Africa. | Does not fit PCC-framework: Supplies and drugs, not medical device | <https://dx.doi.org/10.1016/j.socscimed.2018.01.008> |
| Madhlambudzi 2019 | Stakeholder identification and salience in purchasing: An empirical study from UK hospitals | Exclusion criteria: Systematic review | <https://dx.doi.org/10.1504/IJHTM.2019.104933> |
| Maia 2021 | Decision-making processes for magnetic resonance imaging technology procurement: Decision-makers perspectives | Does not fit PCC-framework: MR scanner focus, too narrow. | <https://dx.doi.org/10.1159/000520543> |
| Malcolm 2015 | Prevalence and Perioperative Outcomes of Off-Label Total Hip and Knee Arthroplasty in the United States, 2000-2010. | Not generalizable | <https://dx.doi.org/10.1016/j.arth.2015.05.020> |
| Malik 2019 | PMD31 SWIPING RIGHT ON MEDICAL TECHNOLOGY; AN ANALYSIS OF DRUG VS. DEVICE HTA APPROVALS IN CANADA | Does not fit PCC-framework | <https://dx.doi.org/10.1016/j.jval.2019.04.1028> |
| Mamas 2012 | Longitudinal stent deformation: insights on mechanisms, treatments and outcomes from the Food and Drug Administration Manufacturer and User Facility Device Experience database. | Not generalizable | <https://dx.doi.org/10.4244/EIJV8I2A33> |
| Mantuano 2016 | Regional market access pathways for diagnostics in Italy | Not generalizable | Value in Health / 2016;19(7):A703 |
| Mao 2021 | Food and Drug Administration Safety Communication on the Use of Transvaginal Mesh in Pelvic Organ Prolapse Repair Surgery: The Impact of Social Determinants of Health. | Does not fit PCC-framework | <https://dx.doi.org/10.1097/SPV.0000000000000863> |
| Marcaccio 2022 | Disparities in reporting and representation by sex, race, and ethnicity in endovascular aortic device trials. | Not generalizable | <https://dx.doi.org/10.1016/j.jvs.2022.05.003> |
| Matheny 2006 | Monitoring device safety in interventional cardiology. | Not generalizable | Journal of the American Medical Informatics Association : JAMIA / 2006;13(2):180-7 |
| Michels 2022 | Expectations, Needs And Challenges Of Setting Up An International Collaboration On Horizon Scanning For Medical Devices | Does not fit PCC-framework | <https://dx.doi.org/10.1017/S0266462322000885> |
| Miech 2021 | The Participant-Reported Implementation Update and Score (PRIUS): A Novel Method for Capturing Implementation-Related Data Over Time. | Does not fit PCC-framework: Not medical device specific | <https://dx.doi.org/10.3791/61738> |
| Milewa 2008 | Representation and legitimacy in health policy formulation at a national level: perspectives from a study of health technology eligibility procedures in the United Kingdom. | Exclusion criteria: Editorial/Opinion/Comment paper | Health policy (Amsterdam, Netherlands) / 2008;85(3):356-62 |
| Morrison 2016 | Innovations in modeling and simulation: Patient-centered healthcare | Does not fit PCC-framework:  Conference discussion only | <https://dx.doi.org/10.1007/s10439-016-1710-7> |
| Morrison 2017 | The Role of Computational Modeling and Simulation in the Total Product Life Cycle of Peripheral Vascular Devices. | Exclusion criteria: Editorial/Opinion/Comment paper | <https://dx.doi.org/10.1115/1.4035866> |
| Moshi 2020 | Development of a health technology assessment module for evaluating mobile medical applications. | Does not fit PCC-framework:  Not on approval or implementation | <https://dx.doi.org/10.1017/S0266462320000288> |
| Moyimane 2017 | Experiences of nurses on the critical shortage of medical equipment at a rural district hospital in South Africa: A qualitative study | Does not fit PCC-framework | <https://dx.doi.org/10.11604/pamj.2017.28.100.11641> |
| Neeser 2017 | New European medical device directive (MDD) comes into effect in 2020: Chances and consequences for stakeholders | Not generalizable | <https://dx.doi.org/10.1016/j.jval.2017.08.1112> |
| Ogawa 2014 | Post-purchase reassessment and improvement of neuroendoscope holder: importance of physician-manufacturer communication | Does not fit PCC-framework | <https://dx.doi.org/10.3233/THC-140781> |
| Oikonomidis 2019 | Material failure in dynamic spine implants: are the standardized implant tests before market launch sufficient?. | Not generalizable | <https://dx.doi.org/10.1007/s00586-019-05880-y> |
| Olberg 2017 | Scientific Evidence in Health Technology Assessment Reports: An In-Depth Analysis of European Assessments on High-Risk Medical Devices. | Does not fit PCC-framework | <https://dx.doi.org/10.1016/j.jval.2017.05.011> |
| Palojoki 2019 | Reporting medical device safety incidents to regulatory authorities: An analysis and classification of technology-induced errors | Does not fit PCC-framework | <https://dx.doi.org/10.1177/1460458217720400> |
| Polisena 2020 | Use of real-world data and evidence for medical devices: a qualitative study of key informant interviews. | Does not fit PCC-framework: Interviews about how stakeholders can use real-life data, not directly on approval. | <https://dx.doi.org/10.1017/S0266462320000859> |
| Portefaix 2019 | Clinical evaluation of medical devices: Proposition for a strategy based on decision trees | Does not fit PCC-framework: Not an empirical study | <https://dx.doi.org/10.1111/fcp.12470> |
| Price 2008 | The off- versus on-label use of medical devices in interventional cardiovascular medicine: Clarifying the ambiguity between regulatory labeling and clinical decision-making, Part 1: PCI | Exclusion criteria: Editorial/Opinion/Comment paper | <https://dx.doi.org/10.1002/ccd.21697> |
| Rassen 2012 | Using high-dimensional propensity scores to automate confounding control in a distributed medical product safety surveillance system. | Does not fit PCC-framework: Not an empirical study | <https://dx.doi.org/10.1002/pds.2328> |
| Ramanathan 2024 | Safely Introducing New Surgical Devices in Minimally Invasive Gynecologic Surgery: An Argument for Use of the IDEAL Framework. | Not generalizable | <https://dx.doi.org/10.1016/j.jmig.2023.10.015> |
| Resnic 2017 | Registry-based prospective, active surveillance of medical-device safety | Not generalizable | <https://dx.doi.org/10.1016/j.jvs.2017.08.004> |
| Ridout 2022 | Piloting scale up of CRADLE into routine maternity care in Sierra Leone | Not generalizable | <https://dx.doi.org/10.1111/1471-0528.1_17178> |
| Rising 2015 | Characteristics of pivotal trials and FDA review of innovative devices. | Not generalizable | <https://dx.doi.org/10.1371/journal.pone.0117235> |
| Roginiel 2018 | Evidence supporting FDA approval and CMS national coverage determinations for novel medical products, 2005 through 2016: A cross-sectional study | Does not fit PCC-framework | <https://dx.doi.org/10.1097/MD.0000000000012715> |
| Ross 2010 | The patient's perspective on evidence needed to support the regulation and adoption of new surgical devices: Preliminary results from a qualitative pilot study | Does not fit PCC-framework | <https://dx.doi.org/10.1177/1740774510374795> |
| Rummel 2017 | European assessments of medical devices: Avenues for improvement | Does not fit PCC-framework | <https://dx.doi.org/10.1017/S0266462317001416> |
| Russell 2006 | Off-label use: an industry perspective on expanding use beyond approved indications. | Exclusion criteria: Systematic review | Journal of interventional cardiology / 2006;19(5):432-8 |
| Saleem 2015 | Understanding barriers and facilitators to the use of Clinical Information Systems for intensive care units and Anesthesia Record Keeping: A rapid ethnography. | Does not fit PCC-framework | <https://dx.doi.org/10.1016/j.ijmedinf.2015.03.006> |
| Saleem 2015 | Overview of high-risk medical device innovation in gastroenterology from 2000-2014: Enhancing the pipeline | Not generalizable | <https://dx.doi.org/10.14309/00000434-201510001-02249> |
| Sapirstein 1994 | The role of clinical trials in the Food and Drug Administration approval process for cardiovascular devices. | Exclusion criteria: Editorial/Opinion/Comment paper | Circulation / 1994;89(4):1900-2 |
| Schnell-Inderst 2022 | MT4 Study Design Recommendations in ISO Standards for High-Risk Medical Devices: A Systematic Review of the HORIZON2020 Core-MD Project | Does not fit PCC-framework | <https://dx.doi.org/10.1016/j.jval.2022.09.1878> |
| Schnell-Inderst 2018 | Recommendations for primary studies evaluating therapeutic medical devices were identified and systematically reported through reviewing existing guidance. | Exclusion criteria: Systematic review | <https://dx.doi.org/10.1016/j.jclinepi.2017.10.007> |
| Seltzer 2017 | Use of endpoint adjudication to improve the quality and validity of endpoint assessment for medical device development and post marketing evaluation: Rationale and best practices. A report from the cardiac safety research consortium. | Exclusion criteria: Editorial/Opinion/Comment paper | <https://dx.doi.org/10.1016/j.ahj.2017.05.009> |
| Sengupta 2020 | Outcomes Before and After the Recall of a Heart Failure Pacemaker. | Not generalizable | <https://dx.doi.org/10.1001/jamainternmed.2019.5171> |
| Seo 2019 | PMD44 PRICING AND REIMBURSEMENT, AND MARKET ACCESS OF THERAPEUTIC MATERIALS (MEDICAL DEVICES/DIAGNOSTICS/BIOMARKERS) IN SOUTH KOREA | Exclusion criteria: Reimbursement | <https://dx.doi.org/10.1016/j.jval.2019.09.1457> |
| Shah 2006 | User involvement in healthcare technology development and assessment: structured literature review. | Exclusion criteria: Systematic review | International journal of health care quality assurance incorporating Leadership in health services / 2006;19(6-7):500-15 |
| Siebert 2002 | Health technology assessment for medical devices in Europe. What must be considered. | Could not be retrieved | International journal of technology assessment in health care / 2002;18(3):733-40 |
| Silva 2011 | Health technology diffusion in developing countries: a case study of CT scanners in Brazil. | Does not fit PCC-framework | <https://dx.doi.org/10.1093/heapol/czq076> |
| Smith 2019 | Defining the clinician's role in early health technology assessment during medical device innovation - a systematic review. | Does not fit PCC-framework & Exclusion criteria: Systematic review | <https://dx.doi.org/10.1186/s12913-019-4305-9> |
| Smith 2020 | Industry ties and evidence in public comments on the FDA framework for modifications to artificial intelligence/machine learning-based medical devices: a cross sectional study. | Does not fit PCC-framework | <https://dx.doi.org/10.1136/bmjopen-2020-039969> |
| Song 2018 | Retrospective analysis of RF heating measurements of passive medical implants. | Not generalizable | <https://dx.doi.org/10.1002/mrm.27346> |
| Sorenson 2013 | Decison making under uncertainty: Coverage with evidence development in the context of medical devices | Does not fit PCC-framework | <https://dx.doi.org/10.1016/j.jval.2013.08.035> |
| Sorenson 2011 | Medical technology procurement in Europe: a cross-country comparison of current practice and policy. | Does not fit PCC-framework: How procurement is organized, not about the underlying factors for decision-makers or efficacy/safety requirements. | <https://dx.doi.org/10.1016/j.healthpol.2010.08.001> |
| Speed 2023 | Otologic use of porcine small intestinal submucosal graft (biodesign): A MAUDE database review. | Not generalizable | <https://dx.doi.org/10.1016/j.amjoto.2023.103961> |
| Spitzenberger 2014 | Laboratory analysis for clinical studies-Regulatory requirements for testing of medicinal products and medical devices according to national and European legislation | Does not fit PCC-framework: About law | <https://dx.doi.org/10.1515/cclm-2014-0863> |
| Sprange 2012 | The NICE Medical Technologies Evaluation Programme (MTEP): manufacturer submission challenges. | Does not fit PCC-framework | <https://dx.doi.org/10.1258/jrsm.2012.120042> |
| Steinemann 2005 | Over-the-counter decorative contact lenses: Cosmetic or Medical Devices? A Case Series. | Not generalizable | Eye & contact lens / 2005;31(5):194-200 |
| Stordeur 2013 | [Introduction of innovative high-risk medical devices in Europe: are clinical efficacy and safety guaranteed?]. | Exclusion criteria: Opinion/Comment/Editorial | <https://dx.doi.org/10.1016/j.respe.2012.08.004> |
| Teames 2020 | Characterization of Device-Related Malfunction, Injury, and Death Associated with Using Elastomeric Pumps for Delivery of Local Anesthetics in the US Food and Drug Administration MAUDE Database. | Not generalizable | <https://dx.doi.org/10.2147/DHPS.S280006> |
| Tomic 2018 | The review and classification of the most commonly used surgical sutures | Does not fit PCC-framework | <https://dx.doi.org/10.1136/ejhpharm-2018-eahpconf.38> |
| Tong 2022 | Adverse Events Associated With Ossicular Prostheses: Utility of a Federal Database. | Not generalizable | <https://dx.doi.org/10.1097/MAO.0000000000003408> |
| Trippoli 2022 | Relationship Between Price and Diagnosis-Related Group Tariff for Medical Devices Assessed by a Regional Health Technology Assessment Committee. | Does not fit PCC-framework: Related to pricing of diagnosis-related group for medical devices | <https://dx.doi.org/10.7759/cureus.23092> |
| Triulzi 2017 | Public hospital supply chain: Current view and critical issues in Italy | Does not fit PCC-framework: Regarding the supply chain | <https://dx.doi.org/10.1016/j.jval.2017.08.660> |
| Triulzi 2019 | Demand side and supply side of healthcare supply chain | Does not fit PCC-framework: Regarding the supply chain | International Journal of Technology Assessment in Health Care / 2019;35(Supplement 1):66-67 |
| Triulzi 2016 | Development of an objective feedback score for the evaluation of drug and medical device manufacturers/distributors as an additional parameter to be considered in public procurement | Does not fit PCC-framework: Development of a score, but not a concrete assessment of the balance between pros and cons. | <https://dx.doi.org/10.1136/ejhpharm-2016-000875.259> |
| Tsougranis 2020 | The Vascular Implant Surveillance and Interventional Outcomes (VISION) Coordinated Registry Network: An effort to advance evidence evaluation for vascular devices. | Exclusion criteria: Opinion/Comment/Editorial | <https://dx.doi.org/10.1016/j.jvs.2020.04.507> |
| Ullman 2019 | Skin complications associated with vascular access devices: A secondary analysis of 13 studies involving 10,859 devices. | Not generalizable | <https://dx.doi.org/10.1016/j.ijnurstu.2018.10.006> |
| Ulucanlar 2013 | Technology identity: the role of sociotechnical representations in the adoption of medical devices. | Does not fit PCC-framework: Regarding political organization | <https://dx.doi.org/10.1016/j.socscimed.2013.09.008> |
| Vaduganathan 2015 | Contemporary Cardiovascular Device Clinical Trials (Trends and Patterns 2001 to 2012) | Exclusion criteria: Systematic review | <https://dx.doi.org/10.1016/j.amjcard.2015.03.062> |
| Varma 2010 | Use of patient-reported outcomes in medical product development: a report from the 2009 NEI/FDA Clinical Trial Endpoints Symposium. | Does not fit PCC-framework | <https://dx.doi.org/10.1167/iovs.10-5627> |
| Viceconti 2017 | In silico assessment of biomedical products: The conundrum of rare but not so rare events in two case studies. | Not generalizable | <https://dx.doi.org/10.1177/0954411917702931> |
| Vincent 2017 | Bags, batteries and boxes: A qualitative interview study to understand how syringe drivers are adapted and used by healthcare staff. | Does not fit PCC-framework | <https://dx.doi.org/10.1016/j.apergo.2017.04.012> |
| Vincent 2017 | How do health service professionals consider human factors when purchasing interactive medical devices? A qualitative interview study. | Does not fit PCC-framework | <https://dx.doi.org/10.1016/j.apergo.2016.08.025> |
| Virk 2020 | A cross-sectional analysis of 1347 complications for cervical disc replacements from medical device reports maintained by the United States Food and Drug Administration | Not generalizable | <https://dx.doi.org/10.1016/j.spinee.2020.09.005> |
| Vogler 2022 | Assessing, Pricing and Funding Point-of-Care Diagnostic Tests for Community-Acquired Acute Respiratory Tract Infections-Overview of Policies Applied in 17 European Countries | Does not fit PCC-framework: Related to pricing of point of care tests | <https://dx.doi.org/10.3390/antibiotics11080987> |
| Waetjen 2015 | Obstacles to Studying Emerging Technologies. | Exclusion criteria: Opinion/Comment/Editorial | <https://dx.doi.org/10.1097/AOG.0000000000000914> |
| Wallace 2021 | Vaginal energy-based devices: characterization of adverse events based on the last decade of MAUDE safety reports | Not generalizable | <https://dx.doi.org/10.1097/GME.0000000000001661> |
| Wang 2016 | Trend Analysis of FDA Warning Letters Issued to Medical Products About Violations to Current Good Manufacturing Practices (CGMP) Between 2007 and 2014. | Does not fit PCC-framework: Not device specific and is not within our scope | <https://dx.doi.org/10.1177/2168479015619201> |
| Wang 2020 | Toward standardized premarket evaluation of computer aided diagnosis/detection products: insights from FDA-approved products. | Do not fit PCC-framework | <https://dx.doi.org/10.1080/17434440.2020.1813566> |
| Wang 2023 | Endovascular Aneurysm Repair Devices as a Use Case for Postmarketing Surveillance of Medical Devices. | Not generalizable | <https://dx.doi.org/10.1001/jamainternmed.2023.3562> |
| Wong 2020 | PMD12 Key Considerations of VALUE Assessment Frameworks for Medical Devices and Diagnostics in ASIA Pacific | Does not fit PCC-framework | <https://dx.doi.org/10.1016/j.vhri.2020.07.318> |
| Woo 2014 | Postmarketing safety of biologics and biological devices. | Exclusion criteria: Systematic review | <https://dx.doi.org/10.1016/j.spinee.2013.09.056> |
| Wu 2021 | How medical AI devices are evaluated: limitations and recommendations from an analysis of FDA approvals | Does not fit PCC-framework | <https://dx.doi.org/10.1038/s41591-021-01312-x> |
| Ye 2019 | Psychosocial Factors Affecting Artificial Intelligence Adoption in Health Care in China: Cross-Sectional Study. | Does not fit PCC-framework | <https://dx.doi.org/10.2196/14316> |
| Zannad 2017 | Role of Payers in the Development of Cardiovascular Therapeutics: Misalignment Between Approval and Reimbursement | Exclusion criteria: Opinion/Comment/Editorial | <https://dx.doi.org/10.1016/j.jacc.2017.10.027> |
| Zhang 2019 | Design and analysis of high-risk medical device clinical trials for diabetes monitoring and treatment: A review | Not generalizable | <https://dx.doi.org/10.1097/JP9.0000000000000030> |
| Ziegler 2021 | A Modular Approach to Combine Postmarket Clinical Follow-Up Studies and Postmarket Surveillance Studies. | Does not fit PCC-framework & Exclusion criteria: Systematic review | <https://dx.doi.org/10.1055/s-0041-1735165> |
| Zhu 2022(a) | Landscape of Oncology-Specific, FDA-Approved, Artificial Intelligence and Machine Learning-Enabled Medical Devices | Does not fit PCC-framework | <https://dx.doi.org/10.1016/j.ijrobp.2022.07.1433> |
| Zhu 2022(b) | The 2021 landscape of FDA-approved artificial intelligence/machine learning-enabled medical devices: An analysis of the characteristics and intended use. | Does not fit PCC-framework | <https://dx.doi.org/10.1016/j.ijmedinf.2022.104828> |
| “Health Quality Ontario” 2018 | Ontario health technology assessment series: Continuous monitoring of glucose for type 1 diabetes: A health technology assessment | Not generalizable | Ontario Health Technology Assessment Series / 2018;18(2):1-160 |
|  |  |  |  |
