# Supplementary material for: Scoping review on regulation, implementation and postmarket surveillance of medical devices
Source: PLoS One. 2025 May 30;20(5):e0325250. doi: 10.1371/journal.pone.0325250 (PMC12124561; doi:10.1371/journal.pone.0325250)
Supplement: Attachment — Submitted filename: Plos one Feb.docx [file pone.0325250.s004.docx]

The manuscript provides valuable insights into the current state of medical device regulation, highlighting the gaps in evidence quality and the challenges faced by regulatory agencies and healthcare providers. While the study is well-organized, several areas require further clarification and detail. I hope you find my comments helpful. Some specific items for clarification are outlined below**:**

- Introduction: The opening sentence could be strengthened by briefly mentioning the challenges faced in medical device regulation (e.g., variability in approval standards, post market surveillance issues) before delving into statistics or regulatory processes.
- Introduction: the explanation of the FDA’s approval process can be improved. Focus on the core differences between the more rigorous PMA pathway and the 510(k) process.
- Introduction: Explicitly highlight the research gap this review is trying to fill
- Methods: The rationale for excluding secondary papers is clear, but the use of "secondary papers" might be confusing. It could be useful to explicitly define this term or rephrase it to avoid ambiguity.
- Methods: The mention of the review transitioning between being classified as a "scoping" or "mapping" review is interesting but could be simplified to avoid unnecessary detail in this section. Condense it to: "We ultimately settled on a scoping review due to the nature of the studies included."
- Methods: Instead of using both "categorizing" and "mapping," stick to one term for consistency.
- Results: The process of identifying and including studies is clearly described, but the phrasing in some places can be simplified. consider: "The search identified 4,904 records, of which 3,862 titles/abstracts were screened after de-duplication."
- Results: The section on post market surveillance contains several useful details but could be more concise. Some sentences are a bit repetitive, especially when describing similar databases or trends.
- Results: Some phrases could be streamlined to avoid redundancy. For example, instead of repeating "two studies" or "three studies," consider summarizing the findings from these studies collectively.
- Discussion: When introducing the IDEAL-D framework, it's important to explain its purpose and why it hasn't been widely implemented.
- Discussion: While evidence gaps are a concern, the section does not explore the nuances of why certain devices might still be implemented without enough data (e.g., urgency, limited alternatives, etc.).
- Discussion: While the discussion mentions that regulatory agencies should improve their evidence requirements, it doesn’t explore the potential consequences of these improvements in depth. What are the practical, ethical, and logistical challenges of improving evidence standards? These could be explored further.
- Limitations: Can be expanded.
  - There could be a potential selection bias in the included studies, as many of them relied on FDA databases, and only a few included studies from the EU or other regions. This geographical and methodological limitation may affect the generalizability of the results.
  - The quality of the data used in the included studies varied, particularly in relation to database-based research. Issues such as missing data, underreporting, and potential inaccuracies in the FDA’s MAUDE database could have impacted the findings.
  - Medical devices vary widely in their complexity, use, and risk profiles (from simple bandages to life-saving pacemakers). This heterogeneity could have led to difficulties in generalizing the findings across different types of devices and in assessing the adequacy of evidence for such a diverse range.
  - There may be publication bias in the included studies, as studies with negative or inconclusive findings might be less likely to be published or included in databases. This could result in an overrepresentation of studies with positive or supportive findings on regulatory practices.
  - There may be publication bias in the included studies, as studies with negative or inconclusive findings might be less likely to be published or included in databases. This could result in an overrepresentation of studies with positive or supportive findings on regulatory practices.
- Conclusion: Consider stressing the importance of international collaboration in medical device regulation and surveillance. Since the regulatory practices in the FDA and EU differ, there may be opportunities for harmonization to improve both systems.
- Conclusion: The recommendation to "strengthen evidence" is useful, but it could be more specific. For example, what types of evidence are most critical (e.g., long-term efficacy, post-market surveillance data)? Also, you could discuss how these improvements would directly impact patient safety, healthcare costs, and device innovation.
